# Obstetric transition: trends in maternal mortality in Somalia over the last two decades

**DOI:** 10.1093/inthealth/ihad121

**Published:** 2024-01-19

**Authors:** Ahmed Aweis, Abdirahman H Osoble, Suleyman A Mohamed, Abdulkadir Aweis

**Affiliations:** Department of Public Health, Benadir University, Mogadishu Somalia; Department Gynaecology and Obstetric, Osoble Hospital, Mogadishu Somalia; Department of Surgery, Kalkaal Hospital, Mogadishu Somalia; Department of Research, Bidhaamiye Centre for Social Development and Rebuilding, Mogadishu Somalia

**Keywords:** maternal health, maternal mortality ratio, obstetric transition, Somalia

## Abstract

**Background:**

This paper sheds light on the trends of the maternal mortality ratio (MMR) and obstetric transition in Somalia over the last two decades.

**Methods:**

This is a descriptive study comparing aggregate secondary data from the 2006 Multiple Indicator Cluster Survey and the 2020 Somali Health and Demographic Survey to show the transition.

**Results:**

A 44% reduction of the MMR from 1044 to 692 per 100 000 live births was observed comparing the two surveys.

**Conclusions:**

Somalia has moved from stage I to stage II of the obstetric transition pathway spectrum and there is optimism that the ongoing strengthening of the health system is paying off.

## Introduction

In global reporting of maternal mortality trends, it was shown that, during the first 5 y of the sustainable development goals (SDGs), there was not much reduction in the estimates of maternal mortality rates. In 2020, it was estimated that 800 women die from a maternal cause every day globally. With these rates, there is no indication that the global target of an SDG of <70 maternal death per 100 000 live births can be reached by 2030.^1^ The inequality in maternal survival across the regions and countries of the world is very large, with 70% of global maternal deaths occurring in sub-Saharan Africa. In Somalia, a sub-Saharan African country, one in 20 women who reach reproductive age die from pregnancy-related complications before reaching the end of their childbearing years, making it a country with one of the highest maternal mortality rates in the world.^2^ Limited access and utilisation of high impact interventions such as antenatal care (ANC), safe birth deliveries by skilled birth attendants and institutional delivery, and a low contraceptive prevalence rate, are believed to contribute to the high maternal mortality in Somalia.^2^ The irony of the tragic event of maternal death is that it is preventable and that there are effective tools to combat it. But because of inequalities across the globe, maternal mortality is very high in low-resource settings. Studies have shown that maternal death is not only tragic for the deceased but that it also has a profound impact on newborns and infants, as well as the education of the children and livelihood of the family.^3^ Somalia has observed a slight improvement in maternal health indicators and a reduction of maternal mortality over the last two decades.^2,4^ This is consistent with trends around the world, which show that countries are gradually moving from patterns of high to low maternal mortality,^5^ as well as transitioning from the natural course of pregnancy and childbirth to the institutionalisation of obstetric care, rising rates of obstetric interventions and, finally, overmedicalisation, a phenomenon known as obstetric transition.^5^

This concept is comparable with the demographic and epidemiologic transitions that inevitably took place after progress and development by societies in the last century.^5^ Obstetric transition was proposed to understand the dynamic process of maternal mortality reduction, and it functions as a framework to explain the coexistence of different strategies for reducing maternal mortality, which can inform national and global policies and programmes.^5^

The Somali Health and Demographic Survey (SHDS), which was conducted in 2020, did not emphasise the progress made in the maternal health indicators compared with what was observed in the Multiple Indicator Cluster Survey (MICS), nor did it mention the obstetric transition that the country has undergone.

Therefore, we feel there is a need to review the obstetric transition that has taken place in Somalia over the last two decades.

## Materials and Methods

### Study design

This is a descriptive study that compares aggregate secondary data from two nationwide surveys that were conducted in the last two decades, namely, the MICS conducted in 2006^4^ and the SHDS, which was conducted in 2020.^2^

### Sampling and sample size

A multistage, stratified cluster sampling approach was used in the selection of a nationally representative sample of 5969 households in MICS 2006 and 15 826 households in SHDS 2020 A of which were surveyed. Both surveys provided data on health and demographic characteristics, including mortalities, fertilities and other basic maternal and child health indicators from rural, nomadic and urban populations in the country.

### Data management

In this paper we show the trends and progress made, as well as the obstetric transition in maternal health indicators from 2006 to 2020, using the obstetric transition stages^5^ consisting of five stages. Stage I of the obstetric transition is when the maternal mortality ratio (MMR) is >1000 maternal deaths per 100 000 live births and most women experience a situation similar to the natural history of pregnancy and childbirth, while stage II of the obstetric transition is when the MMR is 300–999 maternal deaths per 100 000 live births. For both of these two stages, demand for and access to lifesaving maternal health services are issues of concern,^5^ and several low- and middle-income countries are in these two stages of the obstetric transition pathway.^5^ Stages III and IV of the obstetric transition pathway correspond to countries with improved access and with a MMR of 50–299 and <50 maternal deaths per 100 000 live births, respectively. Finally, stage V, which at the moment is an aspirational and largely theoretical stage, represents when all preventable maternal mortalities are avoided.^5^

## Results

Table 1 shows a comparison of the data of the MICS 2006 and SHDS 2020, where the estimated MMR is approximately 1044 maternal deaths per 100 000 live births in MICS 2006 compared with a reduction of nearly 44%, consisting of an estimated MMR of 692 maternal deaths per 100 000 live births in SHDS 2020. In reference to the obstetric transition stages,^5^ the decreased MMR lies within 300–999 maternal deaths, which places the country in stage II of the obstetric transition pathway.

**Table 1.** Comparison of maternal health indicators from MICS 2006 and SHDS 2020

Variable	*MICs 2006	**SHDS 2020
MMR	1044 per 100 000 live births	692 per 100 000 live births
ANC	26%	31%
Institutional delivery	9%	21%
Skilled attendants at deliveries	33%	32%
Postnatal care	12%	11%
Modern contraception prevalence	1%	0.9%
Total fertility rate	6.7 children per woman	6.9 children per woman

*MICS 2006: Multiple Indicator Cluster Survey conducted in 2006 across Somalia.

**SHDS 2020: Somali Health and Demographic Survey conducted in 2020 across Somalia.

In addition, MICS 2006 reported that 9% of mothers in labour delivered in health facilities compared with 21% of mothers delivering in hospitals in SHDS 2020; on the other hand, it was shown that 26% and 31% of women aged 15–49 y received ANC delivered by skilled healthcare workers in MICS 2006 and SHDS 2020, respectively.

Furthermore, we found a pattern of small variations between some indicators in the two surveys; for example, the rate of deliveries conducted by a skilled birth attendant was 33% and 32% in MICS 2006 and SHDS 2020, respectively, whereas for postnatal care the corresponding rates were 12% and 11%. Also, the modern contraceptive prevalence was 1% and 0.9% in MICS 2006 and SHDS 2020, respectively, while the corresponding total fertility rates were 6.7 and 6.9 children per woman.

## Discussion

Based on the surveys conducted in the last two decades by the Somali government and the United Nations,^2,4^ it can be seen that the progress made is not trivial. Maternal mortality was reduced by 44% from 1044 maternal deaths per 100 000 live births in 2006^4^ to 694 maternal deaths per 100 000 live births in 2020.^2^ Besides the two nationwide surveys conducted in the country, a global MMR point estimate has shown a downward trend of maternal mortality in Somalia. The MMR point estimates were 1097, 1080, 963, 761 and 621 in 2000, 2005, 2010, 2015 and 2020, respectively.^1^ This is comparable with the worldwide progress in the reduction of MMR globally, as it showed that 446 000 mothers died during pregnancy, while giving birth or 42 days after birth in 2000 compared with 287 000 maternal deaths in 2020.^1^ In other words, the MMR was reduced from 339 maternal deaths per 100 000 live births in 2000 to 223 maternal deaths per 100 000 live births in 2020,^1^ with an average annual rate of reduction of 2.1% in the MMR from 2000 to 2020.

Fragile and conflict-affected countries, such as Nigeria and South Sudan, are still categorised as having extremely high maternal mortality and are still in stage 1 of the obstetric transition pathway, as per the 2020 report.^1^ On the other hand, Rwanda has made good progress, going from 1007 maternal deaths per 100 000 live births in 2000 to 259 in 2020, that is, moving from stage I to stage III of the obstetric transition spectrum.^1^

The data comparison of the two surveys has shown that there has been an improvement in the utilisation of maternal health interventions and a reduction of the MMR in Somalia in the last two decades. This also implies that the country has made a leap from stage 1 to stage 2 in the obstetric transition pathway framework in 15 y.^5^

Although the SHDS and MICS provide a good comparison for observing trends and tracking the progress made in the improvement of maternal health indicators in Somalia over the last two decades, this comparison is not without constraints. The SHDS sample size was threefold that of the number of households sampled and surveyed for the MICS. Furthermore, nomadic people were more prominent in the SHDS than in the MICS. However, these two reports were the only two available with the variables of interest showing the obstetric transition in the country.

In conclusion, some progress has been made in the reduction of maternal mortality in Somalia in the last two decades, by moving to stage 2 of the obstetric transition spectrum.

Despite being slow and insufficient, this brings hope that the country is moving in the right direction, and that the efforts being made, as well as the interventions implemented towards reducing maternal mortality in Somalia are paying off. Many of the other maternal health indicators used to track reductions in maternal mortality have not shown good progress, and significant work is needed to achieve the global targets set for maternal mortalities.

In this regard, there is a need to improve maternal health interventions in the country, such as ANC, institutional delivery by skilled birth attendants and the family planning service. There is also a need to explore more of the factors contributing to the obstetric transition of maternal mortality in Somalia.

## Data Availability

None.

## References

[1] WHO . Trends in maternal mortality 2000 to 2020: Estimates by WHO, UNICEF, UNFPA, World Bank Group and UNDESA/Population Division. 2023.

[2] The Directorate of National Statistics, Ministry of Planning, Investment and Economic Development F, Somalia. G of S. Somali Health and Demographic Survey 2020. 2020.

[3] Bergevin Y, Fauveau V, McKinnon B. Towards ending preventable maternal deaths by 2035. Semin Reprod Med. 2015;33:23–9. 25565508 10.1055/s-0034-1395275

[4] Library M . Somalia—Multiple indicator Cluster Survey 2011, Somaliland. United Nations Child Fund, Somalil Minist Natl Plan Dev. 2016:1–14.

[5] Souza JP, Tunçalp Ö, Vogel JP, et al. Obstetric transition: The pathway towards ending preventable maternal deaths. BJOG. 2014;121(Suppl):1–4. 10.1111/1471-0528.12735 24641529

